# Medical Items

**Published:** 1894-08

**Authors:** 


### MEDICAL ITEMS.

Dr. Charles S. Briggs, of Nashville, will succeed his father, the late Dr. W. T. Briggs, as Professor of Surgery in the Medical Department of Vanderbilt University.

Dr. Emory Lanphear will hereafter conduct the St. Louis *Clinique* "in the interests of the College of Physicians and Surgeons and of the medical profession of the West."

Dr. W. E. Campbell, lately of South Carolina, has located in Atlanta, and entered into partnership with Dr. George Brown. Will practice only diseases of the eye, ear, nose and throat.

Dr. Lewis S. McMurtry, of Louisville, Dr. C. A. L. Reed, of Cincinnati, and Dr. A. L. Hummel, of Philadelphia, are the delegates of the American Medical Association to the British Medical Association which meets in Bristol, England, this month.

The first number (June) of the *Southern Medical Review*, published by Dr. N. J. Phenix, of Houston, Texas, is before us. It contains two or three very good original articles. The remainder of the matter, including the editor's salutatory, is very ordinary. The editor advocates an increase in the number of journals as well as an advance in their standard of excellence. He has increased the number where there is no need for increase, and he has failed to improve the standard in spite of the great desirableness of such improvement.

THE next meeting of the Tri-State Medical Society of Georgia, Alabama and Tennessee will be held in Atlanta, October 9, 10 and 11. It is hoped that there will be a large attendance. President, J. B. S. Holmes, Atlanta; Secretary, Frank Trester Smith, Chattanooga.

---

THE North Carolina Board of Medical Examiners has offered, when complaints are made by candidates who failed to pass, that the questions submitted to the applicant, together with his written answers, should be published, and no one who was rejected has chosen to make the exhibit.

---

INDIGNANT PHYSICIAN—"Man, what have you done? You sent my patient the wrong prescription, and it killed him."

DRUGGIST—"Vell, vat vas der matter mit you? Last veek I send your odder patient der righd berscription, und dot killed him. How can somebody blease sooch a man?"—*Cincin. Med. Journal.*

---

FOOD FOR THOUGHT.—How was it that Marion Sims, Flint, Agnew, Keating, Fordyce Barker, Sir Andrew Clark, Charcot, Billroth, and others, had so much time for literary work? And yet their professional duties were certainly as pressing as any one's we know. It would seem that they felt the necessity of keeping their brains in good working order by writing; and if they thought so, no one could hardly be excused from saying, "Oh! I can find no time for writing." —*Rhode Island Medical Science Monthly.*

